# Mesenchymal Stem Cell-Derived Extracellular Vesicles for Functional Intracellular Delivery: Engineering, Targeting, Cellular Uptake, Biological Barriers, and Therapeutic Translation

**DOI:** 10.3390/cells15211987

**Published:** 2026-10-30

**Authors:** Nuray Üremiş, Muhammed Mehdi Üremiş

**Affiliations:** Department of Medical Biochemistry, Faculty of Medicine, Kahramanmaraş Sütçü İmam University, Kahramanmaraş 46050, Türkiye

**Keywords:** mesenchymal stem cells, extracellular vesicles, drug delivery systems, cargo loading, surface engineering, targeted therapy, clinical translation

## Abstract

**Highlights:**

**What are the main findings?**

- Engineering strategies improve MSC-EV cargo loading, cellular targeting, and therapeutic delivery.
- Cellular uptake alone does not demonstrate functional cargo delivery, which depends on intracellular trafficking, cargo release, and biological activity.

**What is the implication of the main finding?**

- Functional delivery should be evaluated using cargo-specific assays that confirm molecular target engagement and biological responses.
- Clinical translation requires coordinated optimization of EV engineering, therapeutic potency, and manufacturing standardization.

**Abstract:**

Mesenchymal stem cell-derived extracellular vesicles (MSC-EVs) are considered promising biological carriers for biomolecule and drug delivery because of their inherent biological activities, biocompatibility, and capacity to carry a variety of therapeutic cargoes. However, the limited cargo-loading capacity of native EVs, their inefficient delivery to target cells, intracellular trafficking barriers, and limitations in the functional release of their cargo restrict their therapeutic applications. EV engineering encompasses a range of approaches to overcome these limitations, including donor cell modification, endogenous cargo loading and active sorting mechanisms, post-isolation loading methods, surface engineering, and EV–lipid-based hybrid systems. In this review, engineered MSC-EVs are evaluated in terms of cargo loading, targeting, cellular uptake, intracellular trafficking, and functional cargo delivery. We also compare the loading strategies used for nucleic acids, proteins, enzymes, and small molecules, in terms of their advantages, their limitations, and their effect on EV biological activity. The therapeutic applications of MSC-EV-based systems in cardiovascular diseases, wound healing, neurological and musculoskeletal disorders, inflammatory diseases, and cancer are evaluated. The central conclusion of this review is that the therapeutic efficacy of MSC-EVs depends not only on the amount of cargo delivered but also on its delivery to the appropriate target cells, intracellular targeting, and preservation of its biological activity. Accordingly, future EV engineering strategies should aim to simultaneously improve cargo loading, disease-specific targeting, local retention, and cargo stability. Progress on these four fronts is what would make MSC-EVs predictable enough for clinical use.

## 1. Introduction

Extracellular vesicles (EVs) are lipid-bilayer-enclosed structures secreted by cells that mediate intercellular communication by transferring biomolecules to target cells [1]. While their membrane structures help protect the biological molecules they carry in the extracellular environment, EV surface components regulate interactions with target cells and cellular uptake processes [2]. Because they can protect and transport biological cargoes to target cells, EVs are considered natural delivery systems for the intracellular delivery of therapeutic molecules [3]. However, these properties vary depending on the cellular source of the EVs, their molecular composition, production conditions, and the engineering approaches applied [4]. In this context, mesenchymal stem cells (MSCs) have emerged as an important cellular source among the various cell types being investigated for therapeutic EV production [5]. Mesenchymal stem cell-derived extracellular vesicles (MSC-EVs) can reproduce a substantial part of the immunomodulatory, angiogenic, and regenerative paracrine activity of their source cells, through the proteins, lipids, and regulatory RNAs they carry [6]. Their cell-free nature helps overcome some of the limitations associated with the administration of live MSCs, such as cell survival, engraftment, and long-term cell fate [7]. In addition, the ability to obtain MSCs from different tissue sources, expand them in culture, and modulate their molecular properties makes them a suitable source for the development of EV-based therapeutic platforms [8].

The endogenous proteins and regulatory RNAs of MSC-EVs may complement the effects of engineered therapeutic cargoes, providing an additional rationale for their use in regenerative applications. Other EV sources offer different characteristics relevant to therapeutic delivery. HEK293-derived EVs provide a platform for genetic engineering and scalable production, while macrophage-derived EVs possess immune-related properties relevant to inflammatory diseases and therapeutic delivery. RBC-derived EVs have been investigated as carriers for therapeutic RNAs, whereas milk-derived EVs have attracted interest due to their source availability and potential for oral delivery. However, cargo-loading efficiency, targeting specificity, and manufacturing reproducibility cannot be universally ranked across these EV sources, as they depend on cargo type, producer-cell characteristics, engineering strategies, and isolation procedures. Therefore, EV source selection should consider both the intrinsic biological activity of the vesicles and their suitability for the intended therapeutic application (Table 1).

In addition to their native molecular contents, MSC-EVs can also be engineered to carry a variety of therapeutic cargoes [9]. Nucleic acids, including miRNAs [10,11], siRNAs [12] and gene-editing components [13], as well as proteins [14], enzymes [15,16], and small-molecule drugs [17,18], can be loaded into MSC-EVs, and their cargo content can be modulated through modification of the source cells or physical and chemical processing of isolated EVs. In addition, engineering the EV surface enables improved target cell recognition, tissue targeting, and cellular uptake [19]. These advances are transforming MSC-EVs from vesicles that merely carry natural paracrine effects into programmable biological delivery systems capable of delivering specific therapeutic cargoes to target cells [9].

In EV-mediated cargo delivery, vesicular cellular internalization and the subsequent intracellular acquisition of biological functionality by the delivered cargo represent interconnected yet distinct stages of the delivery process [20]. Following cellular uptake, intracellular cargo availability and its interaction with the intended molecular target are among the key steps determining the biological response [20,21]. Therefore, it is important to consider MSC-EV-based delivery as a multistep process extending from cargo loading to the manifestation of biological function in the target cell [20,22]. While current reviews on MSC-EVs have largely focused on cargo loading, engineering strategies, and therapeutic applications [23,24], assessments that link these aspects to the intracellular fate and function of the cargo remain limited. In this review, the existing literature is examined from the perspective of functional intracellular delivery mediated by MSC-EVs. Starting with cargo-loading and surface-engineering approaches, this review follows the cargo through four checkpoints: uptake, endosomal escape, cytosolic availability, and target engagement. In this regard, the review focuses on both the biological barriers encountered by engineered MSC-EVs and their cargoes and the processes that ultimately enable the cargo to exert its biological function in the target cell.

## 2. Engineering MSC-EVs for Functional Cargo Delivery

**Figure 1:**
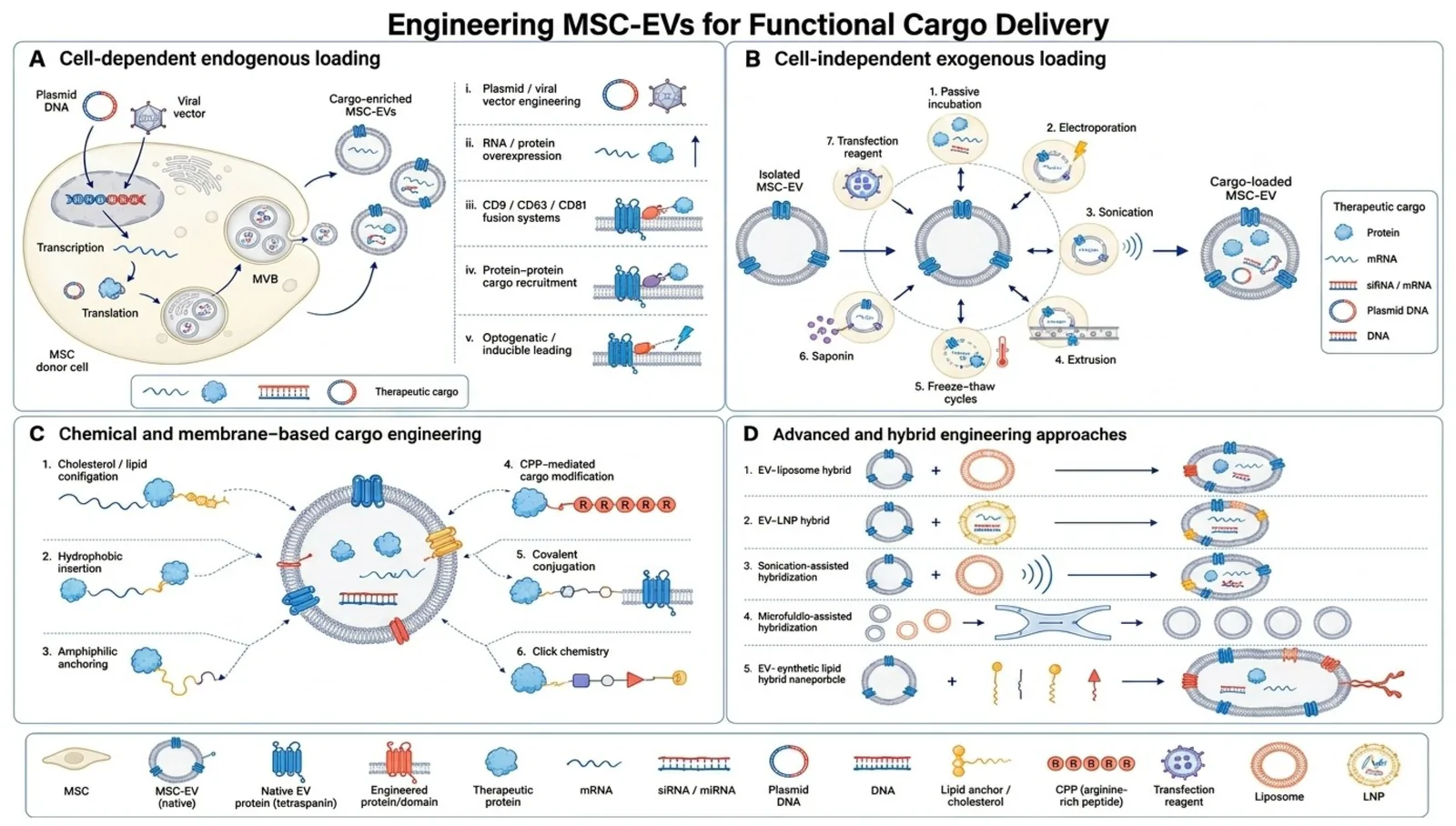
Engineering strategies for functional cargo loading into mesenchymal stem cell-derived extracellular vesicles (MSC-EVs). (**A**) Cell-dependent (endogenous/pre-isolation) loading strategies, in which therapeutic cargoes are incorporated into EVs during biogenesis through donor-cell engineering, including plasmid- or viral vector-mediated expression, RNA or protein overexpression, EV-associated tetraspanin fusion systems, protein–protein interaction-based cargo recruitment, and optogenetic or inducible loading approaches. (**B**) Cell-independent (exogenous/post-isolation) loading of isolated MSC-EVs using passive incubation, electroporation, sonication, extrusion, freeze–thaw cycles, saponin-mediated permeabilization, or transfection reagents. (**C**) Chemical and membrane-based engineering approaches that enhance cargo association with or incorporation into MSC-EVs through cholesterol/lipid conjugation, hydrophobic modification, amphiphilic anchoring, cell-penetrating peptide (CPP)-mediated cargo modification, covalent conjugation, or click chemistry. (**D**) Advanced and hybrid engineering approaches combining MSC-EVs with synthetic lipid-based systems, including EV–liposome and EV–lipid nanoparticle (LNP) hybrids, sonication- or microfluidic-assisted hybridization, and EV–synthetic lipid hybrid nanoparticles. Representative therapeutic cargoes include proteins, mRNA, siRNA/miRNA, plasmid DNA, and DNA. The illustration was conceptually designed by the authors and created using FigureLabs AI (https://figurelabs.ai, accessed on 1 October 2026).

Engineering EVs to carry diverse therapeutic cargoes, including proteins, nucleic acids, and small molecules, enables their use as biological delivery platforms for intracellular cargo delivery [3]. However, the limited cargo-loading capacity of native EVs, variability in their targeting of specific cells and tissues, and constraints on intracellular cargo release remain among the major challenges associated with their therapeutic application [25]. Differences among therapeutic cargoes in terms of molecular size, biological stability, charge, and hydrophilic or hydrophobic properties necessitate the selection of loading strategies according to the physicochemical characteristics of the cargo [25,26]. Therefore, rather than relying on a single loading method applicable to all cargo types, the current literature employs different loading approaches tailored to the properties of the cargo and the intended mode of delivery [26]. Accordingly, various engineering strategies have been developed to enhance MSC-EVs as effective therapeutic cargo delivery systems. These strategies can be classified into four major categories: cell-dependent (endogenous/pre-isolation), cell-independent (exogenous/post-isolation), chemical and membrane-based cargo engineering, and advanced and hybrid engineering approaches (Figure 1; Table 2) [3,25,26].

### 2.1. Cell-Dependent (Endogenous/Pre-Isolation) Loading

Cell-dependent (endogenous/pre-isolation) loading is based on the incorporation of therapeutic cargo into EVs during EV biogenesis [25]. In this approach, donor cells are genetically modified using plasmid DNA or viral vectors to enhance the expression of the target RNA or protein, so that cargo made inside the cell is packaged into EVs by the cell’s own sorting machinery [27]. This approach has been used to enrich UCMSC-EVs with various types of therapeutic RNA [28,29]. For example, overexpression of miR-216 in UCMSCs has been shown to increase miR-216 levels in EVs derived from these cells [28]. Similarly, overexpression of lncRNA HCP5 in UCMSCs resulted in the generation of HCP5-enriched EVs, which were reported to elicit HCP5-mediated biological effects in target cells [29]. However, because increasing cargo expression in donor cells does not always result in sufficient or selective cargo enrichment in EVs, active loading systems have been developed to direct cargo into vesicles during EV biogenesis. To this end, fusion systems that use EV-associated tetraspanins such as CD9, CD63, and CD81 as cargo-targeting platforms, as well as protein–protein interaction-based and inducible loading approaches, have been developed [30,31]. This strategy has been used to optogenetically modify UCMSCs to enhance the loading of eNOS protein into EVs [32]. In this system, the EV-associated protein CD9 and eNOS were coupled to the light-sensitive proteins CIBN and CRY2, respectively; blue light–induced CIBN–CRY2 interaction subsequently promoted the targeting and enrichment of eNOS in EVs.

### 2.2. Cell-Independent (Exogenous/Post-Isolation) Loading

In cell-independent (exogenous/post-isolation) loading approaches, EVs are first isolated from donor cells, after which the therapeutic cargo is loaded directly into the vesicles [33]. The simplest approach is passive loading, which involves incubating EVs with the therapeutic cargo. The applicability of this approach to MSC-EVs has been demonstrated by directly incubating isolated MSC-EVs with curcumin, and the resulting passively loaded curcumin-containing EVs were reported to maintain their stability under both in vitro and in vivo conditions [34]. When the ability of the cargo to cross the EV membrane is limited, active methods such as electroporation, sonication, extrusion, freeze–thaw cycles, and saponin-mediated permeabilization are used to transiently increase membrane permeability. For example, paclitaxel was loaded into EVs isolated from Wharton’s jelly-derived MSCs via electroporation, achieving a loading efficiency of 62% [35]. The applicability of saponin-mediated permeabilization to hMSC-EVs has been demonstrated by incubating isolated EVs with β-glucuronidase in the presence of saponin. This approach was reported to enable encapsulation of the enzyme into EVs while preserving its catalytic activity and vesicle integrity [36]. Transfection reagents have also been used for the cell-independent loading of nucleic acids. In this context, VEGF-A siRNA was loaded into MSC-derived EVs using a transfection reagent, and the delivery of the siRNA to target cells was demonstrated [37]. The use of these exogenous methods provides considerable technical flexibility for loading therapeutic cargoes with diverse physicochemical properties into isolated MSC-EVs.

### 2.3. Chemical, Membrane, and Surface-Targeting Engineering

Beyond conventional endogenous and exogenous loading methods, engineering approaches based on modifying the physicochemical properties of the cargo or the EV membrane have been developed [38]. These strategies may employ cholesterol or lipid conjugation, hydrophobic modifications, and amphiphilic anchoring systems to enhance the interaction of therapeutic molecules with the EV membrane [39,40,41,42]. In this context, siRNA conjugated with cholesterol to increase its hydrophobicity has been shown to be efficiently loaded into Wharton’s jelly-derived UCMSC-EVs through incubation [40]. Similarly, a study evaluating siRNAs conjugated with different lipids reported a clear association between the hydrophobicity conferred by lipid conjugation and the efficiency of siRNA loading into EVs [39]. In another study, modification of the MSC-sEV membrane with cationic amphiphilic ε-polylysine–PEG–DSPE was shown to alter the EV surface charge and enhance cellular uptake by chondrocytes [41]. In addition to hydrophobic modifications and amphiphilic anchoring systems, cell-penetrating peptides (CPPs), covalent conjugation, and click chemistry-based approaches are also employed in EV engineering. CPPs are used as a cargo modification strategy to enhance cargo interaction with the EV membrane and improve loading efficiency. In this context, conjugation of miRNA with the arginine-rich YARA peptide has been shown to enhance its loading into exosomes and delivery to target cells [11]. For covalent surface modification, a study in which alendronate was conjugated to the surface of BM-MSC-derived EVs via copper-free click chemistry demonstrated enhanced bone targeting [43]. Similarly, conjugation of a chondrocyte-affinity peptide (CAP) to the surface of UCMSC-sEVs via click chemistry enhanced EV targeting to chondrocytes and retention within osteoarthritic cartilage [42]. Together, these approaches demonstrate the potential of membrane and surface engineering to improve EV cargo loading, tissue targeting, and cellular interactions. In this process, tissue accumulation, uptake by the intended cell population, and subsequent intracellular cargo release represent distinct but interconnected steps toward functional delivery, each contributing to the development of more effective MSC-EV-based therapeutic platforms.

### 2.4. Advanced and Hybrid Engineering Approaches

Advanced approaches in EV cargo engineering aim to combine the biological properties of natural EVs with the high cargo-loading capacity and engineering flexibility of synthetic lipid-based delivery systems [44]. In this context, EV–liposome hybrids and other lipid-based hybrid nanocarriers have been developed for the delivery of nucleic acids and other therapeutic cargoes [44]. In a study in which BM-MSC-derived exosomes were hybridized with liposomes through freeze–thaw cycles or direct mixing, the resulting hybrid vesicles were shown to deliver a Cas9-GFP plasmid to target cells [45]. Similarly, hybrid vesicles generated by combining MSC-derived exosomes with folate-modified liposomes have been used for paclitaxel delivery [46]. In a more recent study, human MSC-EVs were converted into hybrid lipid nanoparticles with synthetic lipid components using a microfluidic–sonication approach, and the resulting MSC-EV-derived hybrid nanoparticles were shown to encapsulate COL1A1 mRNA and functionally deliver it to tendon progenitor cells [47].

EV–lipid hybrid systems have mainly been investigated to improve the loading of nucleic acids and their delivery into target cells [48]. In addition to lipid-based carriers, EVs have been used in combination with viral vectors and polymeric nanoparticles [48,49]. EV-associated adeno-associated viruses (EV-AAVs) have been developed to reduce the neutralization of AAV capsids by antibodies and improve gene transfer [50]. Experimental studies have shown that EV-associated AAVs can resist antibody neutralization and increase transduction compared with conventional AAV vectors [51]. Polymeric hybrid systems have also been investigated for nucleic acid delivery [52]. For example, EV membrane-coated PLGA nanoparticles have been used to deliver antisense oligonucleotides (GapmeRs). Compared with uncoated PLGA nanoparticles, these hybrid particles showed greater stability in physiological medium and increased cellular uptake [52]. However, hybridization may alter the membrane properties and biological activity of EVs. The extent of these changes depends on the materials and preparation methods used [53,54]. In one study, hybridization with lipid nanoparticles preserved the accessibility and enzymatic activity of EV surface proteins, whereas other EV modification methods have been reported to affect membrane properties and protein activity [54]. Therefore, the biological properties of hybrid EV systems may be affected by the methods used for their preparation.

### 2.5. Advantages and Limitations of EV Engineering Strategies

EV engineering strategies are employed to improve therapeutic cargo-loading efficiency, enhance interactions with target cells, and strengthen the delivery capabilities of EVs [3,38]. However, the advantages of these methods should be evaluated alongside their potential effects on EV structural integrity and biological properties [55,56,57]. Physical loading methods, such as electroporation and sonication, facilitate cargo incorporation by increasing membrane permeability but may also cause alterations in membrane structure, cargo aggregation or degradation, and reduced EV recovery after processing, depending on the experimental conditions [55]. In particular, the formation of nucleic acid aggregates during electroporation can lead to nucleic acids that have not been loaded into EVs being incorrectly identified as EV-associated cargo, resulting in an overestimation of the measured loading efficiency [58]. Similarly, the use of chemical permeabilizing agents, such as saponin, facilitates cargo loading but requires the removal of residual chemical agents after processing [59]. In addition, membrane and surface modifications can improve EV interactions with target cells and tissue targeting, but alterations in native membrane components may also affect EV–cell interactions [56]. Combining EVs with liposomes or lipid nanoparticles offers advantages in terms of cargo-loading capacity and formulation flexibility, while introducing additional challenges related to changes in membrane composition, cargo leakage, and the characterization of hybrid particles [55]. Hybrid EV preparations may contain unmodified EVs, free synthetic nanoparticles, and hybrid particles, making purification and quality control more difficult. Similarly, EV-AAV preparations may contain free AAV vectors that require additional separation steps [50,51]. These products therefore require assessment of particle composition, cargo content, purity, and biological activity [53,54]. These additional processing and purification requirements, together with variations in donor-cell characteristics and manufacturing parameters, can affect EV product purity, yield, and batch-to-batch reproducibility, thereby complicating large-scale manufacturing [55,56,57]. Therefore, MSC-EV engineering should consider not only improvements in cargo-loading efficiency and targeting but also the preservation of EV integrity, cargo stability, post-processing recovery, and biological activity.

## 3. Biological Barriers to Functional Intracellular Delivery

**Figure 2:**
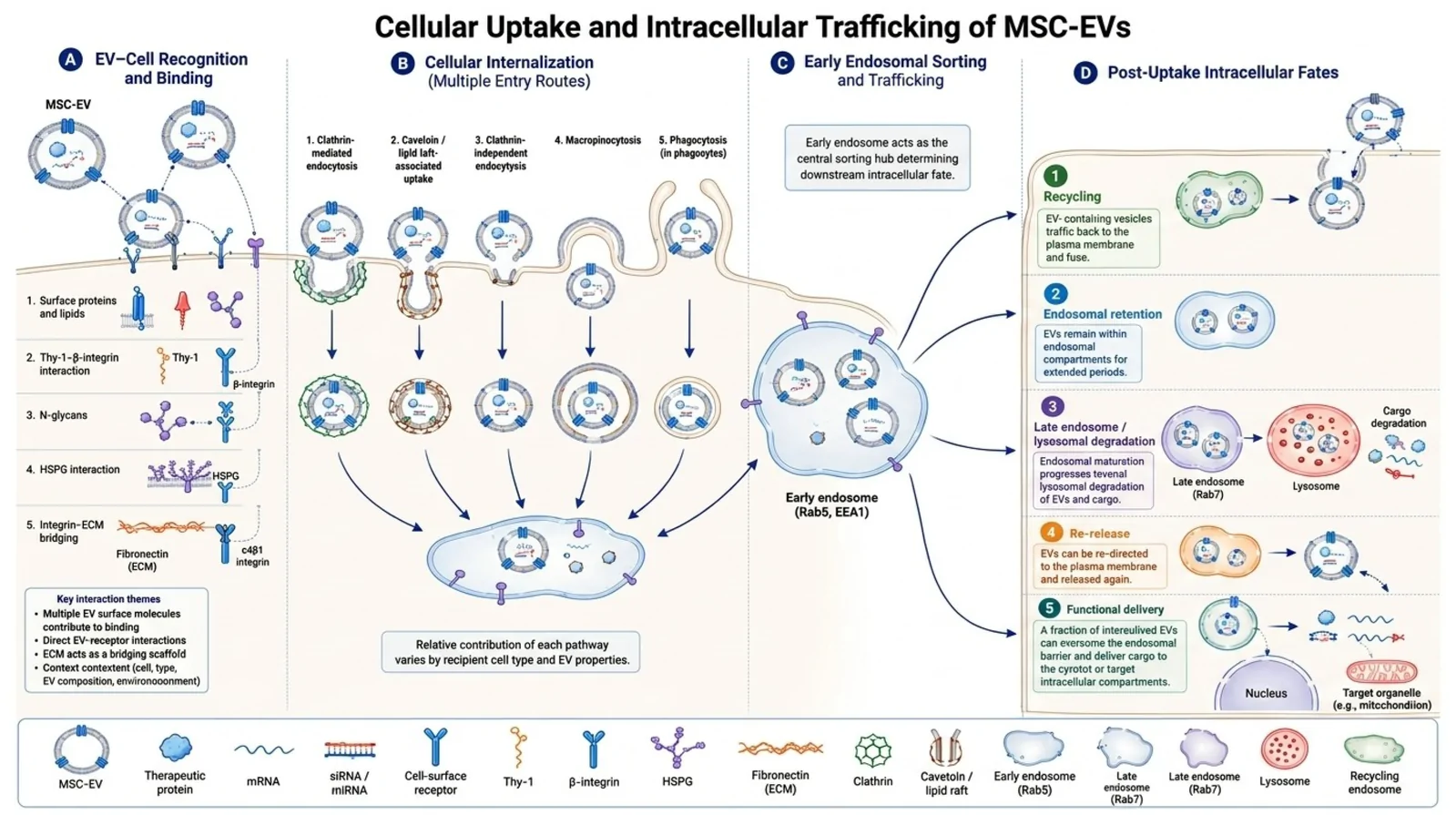
Cellular uptake, intracellular trafficking, and post-internalization fate of mesenchymal stem cell-derived extracellular vesicles (MSC-EVs). (**A**) EV–cell recognition and binding are mediated by multiple surface components and molecular interactions, including EV-associated proteins and lipids, Thy-1–β-integrin interactions, N-glycans, heparan sulfate proteoglycans (HSPGs), and integrin–extracellular matrix (ECM) interactions that can facilitate EV attachment to recipient cells. (**B**) Following cell-surface binding, MSC-EVs can be internalized through multiple, potentially overlapping pathways, including clathrin-mediated endocytosis, caveolin/lipid raft-associated uptake, clathrin-independent endocytosis, macropinocytosis, and phagocytosis, with the relative contribution of each pathway depending on EV properties and recipient cell type. (**C**) Internalized EVs are predominantly trafficked into the endosomal system, where early endosomes serve as central sorting compartments that influence their subsequent intracellular fate. (**D**) Following early endosomal sorting, internalized EVs and their cargoes may undergo recycling to the plasma membrane, remain within endosomal compartments, progress through late endosomes toward lysosomal degradation, be re-released into the extracellular space, or, in a fraction of cases, overcome endosomal confinement and deliver cargo to the cytosol or other intracellular sites of action. These alternative trafficking routes emphasize that cellular uptake alone does not establish functional cargo delivery; therapeutic cargo must become accessible to the intracellular compartment in which it exerts its biological activity. The illustration was conceptually designed by the authors and created using FigureLabs AI (https://figurelabs.ai, accessed on 1 October 2026).

The therapeutic efficacy of engineered MSC-EVs depends not only on the efficient loading of cargo into the vesicles and its delivery to the target tissue, but also on a series of cellular processes that determine whether the delivered cargo becomes biologically functional within recipient cells [21,60]. Following interaction with the cell surface, EVs can be internalized through various mechanisms and subsequently undergo intracellular trafficking, predominantly through endosomal compartments [60]. The route of EV internalization and subsequent intracellular trafficking determines whether the delivered cargo is retained within the endosomal system, directed toward recycling pathways, subjected to lysosomal degradation, or able to reach its intracellular site of action [20]. Particularly for cargoes that require cytosolic access, such as regulatory RNAs, gene-editing components, and proteins targeting intracellular molecules, escape from endosomal confinement represents a critical step in functional intracellular delivery [21,61]. Furthermore, the delivered cargo must retain its molecular integrity and achieve sufficient intracellular bioavailability to interact with its intended target [20,62]. Therefore, the success of MSC-EV-mediated functional delivery should be evaluated in the context of the sequential biological processes extending from cellular uptake to intracellular bioavailability (Figure 2).

### 3.1. Cellular Uptake and Intracellular Trafficking

For MSC-EVs to deliver their therapeutic cargoes to recipient cells, they must first interact with the cell surface, subsequently undergo internalization, and then be directed to appropriate compartments within the intracellular vesicular trafficking network [63]. Current evidence indicates that EV interactions with recipient cells are not governed by a single ligand–receptor system; rather, they are collectively influenced by EV surface composition, the membrane properties of recipient cells, and the extracellular microenvironment [63,64]. In particular, EV surface components such as integrins, tetraspanins, glycoproteins, glycolipids, and phosphatidylserine have been reported to contribute to cellular recognition and binding [64,65]. Proteins and glycans present on the vesicle surface have been shown to play important roles in the interaction of MSC-EVs with recipient cells. In particular, the interaction between Thy-1 and β-integrins has been reported to contribute to the uptake of MSC-EVs by fibroblasts [66], whereas N-glycans on the MSC-EV surface have been shown to be important for their interaction with and uptake by endothelial cells [67]. In the broader EV literature, integrin–ECM interactions have also been shown to contribute to cellular recognition and internalization, with the interaction between α4β1 integrin on recipient cells and fibronectin on the EV surface reported to promote EV uptake and functional RNA transfer [68]. These findings suggest that EV–cell recognition may occur not only through direct EV–receptor interactions but also through tripartite interactions in which the extracellular matrix serves as a molecular bridge.

Although endocytosis is considered a major mechanism for the internalization of EVs bound to the cell surface, no single endocytic pathway has been identified as universally applicable to all EVs. Clathrin-mediated endocytosis, clathrin-independent endocytosis, caveolin/lipid raft-associated uptake, macropinocytosis, and phagocytosis have been demonstrated in different EV–recipient cell systems [69]. The relative contribution of these pathways to MSC-EV internalization varies depending on the recipient cell type [70]. A heparan sulfate proteoglycan-associated, caveolin/lipid raft-dependent mechanism has been demonstrated in the uptake of human MSC-derived EVs by MSCs, monocyte-like cells, and keratinocytes, with inhibition of the caveolar pathway reported to markedly reduce EV internalization [70]. In contrast, both clathrin-mediated endocytosis and macropinocytosis have been shown to contribute to the uptake of UC-MSC-derived exosomes by acute myeloid leukemia cells [71]. A distinct internalization mechanism operates in phagocytic cells, as MSC-derived EVs have been shown to be taken up by macrophages through phagocytosis [72]. In endothelial cells, MSC-sEV internalization has been reported not to depend on a single endocytic mechanism, with multiple pathways potentially contributing in parallel [73]. Thus, the available literature indicates that MSC-EV uptake cannot be attributed to a single endocytic mechanism and that the relative contribution of different internalization pathways may vary depending on EV characteristics and recipient cell type.

EVs internalized through endocytosis generally do not gain direct access to the cytosol but are instead trafficked into the endosomal system [60]. Early endosomes serve as key sorting compartments that determine the subsequent intracellular fate of internalized material [64,74]. At this stage, EVs or EV-associated material may be sorted into recycling pathways for return to the plasma membrane or directed toward downstream compartments during endosomal maturation [20,64]. Although these intracellular trafficking pathways are well characterized in general EV biology, mechanistic insights into the post-internalization trafficking of MSC-EVs remain more limited. Current studies support that MSC-EVs are internalized by different recipient cell types through endocytic mechanisms and can subsequently be trafficked to intracellular vesicular compartments [70,73]; however, the molecular mechanisms governing the sorting of MSC-EVs into recycling or endolysosomal degradation pathways following early endosomal sorting have not yet been fully elucidated.

Endosomal material that is not directed toward recycling pathways may be trafficked to late endosomal and lysosomal compartments during endosomal maturation [64]. During this process, the membrane composition, luminal pH, and molecular regulators of endosomes undergo progressive changes, while the predominantly Rab5-associated identity of early endosomes transitions to a predominantly Rab7-associated profile during late endosomal maturation [75,76]. Studies in the broader EV literature have shown that internalized vesicles can follow this endosomal maturation pathway to reach late endosomes and lysosomes, where they may undergo degradation [20,77]. However, the temporal dynamics of MSC-EV post-uptake trafficking along the Rab5/Rab7 axis and the molecular mechanisms governing their lysosomal targeting in recipient cells have not yet been fully characterized. Nevertheless, not all internalized EVs result in lysosomal degradation; general EV studies demonstrate that, in addition to endosomal retention and degradation, some internalized EVs can be re-released into the extracellular space [20].

Taken together, these findings indicate that cellular uptake alone is not a sufficient measure of successful MSC-EV-mediated therapeutic delivery. Studies in the broader EV literature have shown that post-internalization intracellular trafficking can result in distinct fates, including recycling, endosomal retention, lysosomal degradation, or release back into the extracellular space [20,64,77]. However, a fraction of internalized EVs has also been shown to overcome the endosomal barrier and deliver their cargo to the cytosol or to the intracellular compartment containing its biological target [78,79,80]. Therefore, the route of internalization and subsequent intracellular trafficking are key processes that determine not only the intracellular localization of EVs but also whether the therapeutic cargo becomes functionally accessible. The mechanisms by which EV cargo achieves functional cytosolic access are discussed in the following section.

### 3.2. Functional Intracellular Cargo Delivery: Concepts, Evidence, and Assessment

The ability of MSC-EVs to function as therapeutic carriers depends not only on their internalization by recipient cells but also on the cargo they carry reaching the intracellular compartment where it can exert its biological effect [23,24]. An increasingly accepted view in the current EV literature is that cellular internalization and functional intracellular cargo delivery do not represent the same biological event [60,81]. Indeed, a substantial proportion of internalized EVs are retained within the endosomal system, directed toward lysosomal degradation, or released back into the extracellular space through recycling pathways [20]. Therefore, the demonstration of high levels of EV uptake within a cell does not, on its own, prove that the therapeutic cargo has reached the cytosol or interacted with its molecular target [81]. Accordingly, the fundamental question in evaluating MSC-EV-based delivery systems should not be “Did the EV enter the cell?” but rather “Did the cargo it carried reach the intracellular compartment where it can perform its biological function?” This distinction becomes even more important when the biological characteristics of the therapeutic cargo are taken into consideration, as different classes of cargo exert their functions within distinct intracellular compartments. For example, miRNAs and siRNAs must reach the cytosol to interact with the RNA-induced silencing complex and suppress target gene expression [82], whereas mRNA function depends on its translation in the cytoplasm to initiate protein synthesis [83]. Proteins and enzymes, in turn, must reach the appropriate intracellular environment where they can interact with their target proteins or substrates [84]. In CRISPR/Cas systems, overcoming the endosomal barrier represents only the first step in intracellular delivery; this must be followed by the release of the Cas complex into the cytosol and its subsequent transport to the nucleus to enable genome editing [85]. Therefore, functional intracellular delivery cannot be defined uniformly across all therapeutic cargoes; rather, each cargo type should be evaluated according to its mechanism of biological action.

This perspective also directly influences the interpretation of experimental approaches used in EV studies. Currently, the majority of MSC-EV studies demonstrate cellular uptake using fluorescence microscopy, confocal imaging, or flow cytometry [86]. However, although these methods demonstrate the presence of EVs or their cargo within cells, they do not establish whether the cargo has escaped from endosomal compartments and become biologically accessible [20,81]. This is because the observed fluorescence signal may originate from functional cargo released into the cytosol but may also represent material retained within endosomal or lysosomal compartments [62,87]. Therefore, an increasingly emphasized view in the current literature is that intracellular localization indicates internalization rather than functional delivery [60,87]. At this stage, the experimental approach used to validate functional intracellular delivery should be selected according to the biological characteristics of the cargo being delivered (Table 3). For regulatory RNAs, the strongest evidence of functional delivery is the demonstration of sequence-specific suppression of target gene expression together with the associated molecular or phenotypic changes [62,88]. This has been experimentally demonstrated in several studies using engineered MSC-EVs. For example, miR-124a-enriched MSC-EVs were shown to suppress the expression of the target gene FOXA2 in glioma stem cells, accompanied by reductions in cell proliferation and clonogenic capacity [89]. Similarly, MSC-EV-delivered miR-124 was reported to induce neuronal differentiation by reducing Sox9 expression, in addition to sequence-specific target suppression confirmed using a luciferase reporter assay [90]. Furthermore, PTEN siRNA-loaded bone marrow-derived MSC-EVs (ExoPTEN) were shown to reach the lesion site in a spinal cord injury model, significantly suppress PTEN expression, and promote associated axonal regeneration [91]. Similar to regulatory RNAs, the intracellular detection of mRNA alone is insufficient to demonstrate functional delivery. For mRNA cargoes, functional intracellular delivery requires experimental confirmation that the delivered transcript is translated into a functional protein within the recipient cell [26,92]. In this context, MSC-EVs derived from bone marrow and cord blood were shown to transfer IL-10 mRNA to recipient renal tubular cells. Full-length IL-10 mRNA was detected in the EVs, whereas IL-10 protein was absent from the EV preparations. Following EV treatment, IL-10 mRNA and IL-10 protein were detected in recipient cells by RT-PCR, Western blotting, and immunofluorescence analyses. These findings support the translation of EV-transferred mRNA in recipient cells [93].

For protein and enzyme delivery, the assessment of functional delivery becomes even more critical. Detection of a fluorescently labeled protein within the cell or confirmation of its presence using immunological methods does not establish that the delivered protein remains biologically active [94,95]. Instead, restoration of enzymatic activity, functional rescue, demonstration of substrate conversion, or induction of the expected cellular response provides much stronger evidence of functional delivery [96,97]. Particularly for therapeutic enzymes, biological activity depends not only on their intracellular delivery but also on their ability to retain catalytic activity and interact with their molecular targets within the cell [14,32]. In one study, endothelial nitric oxide synthase (eNOS) was loaded into UCMSC-derived exosomes using the optogenetic EXPLOR system. The engineered exosomes were taken up by endothelial cells and increased nitric oxide levels in diabetic wound tissue compared with unmodified exosomes. They also improved endothelial cell proliferation and migration, promoted angiogenesis, and accelerated wound healing. Although these findings are consistent with the biological effects of eNOS, its release into the cytosol and enzymatic activity within recipient cells were not directly demonstrated [32]. In another study, fibroblast growth factor 1 (FGF1) was loaded into MSC-derived exosomes using a YARA–FGF1–GFP fusion protein. The loaded exosomes were taken up by fibroblasts and significantly increased cell proliferation, migration, and invasion compared with unloaded exosomes and YARA-loaded controls. Although these results support the biological effects of FGF1-loaded exosomes, the release of FGF1 into the cytosol and its intracellular activity were not directly demonstrated [14].

Similarly, in CRISPR/Cas systems, the strongest evidence of functional delivery is not the intracellular detection of Cas9 protein or sgRNA, but the experimental demonstration of genome editing at the target locus, such as indel formation, homology-directed repair, base editing, or target gene inactivation [98]. Nevertheless, the number of studies directly demonstrating the functional intracellular delivery of proteins, RNAs, and particularly CRISPR/Cas components by MSC-EVs remains limited. In contrast, loading platforms developed using engineered non-MSC EVs, including CD63-Com [99], NanoMEDIC [100] and safeEXO [101], have demonstrated the feasibility of EV-mediated functional genome editing. These advances suggest that similar strategies could be adapted to MSC-EV platforms in the future.

Studies involving miRNA, siRNA, mRNA, protein, enzyme, and CRISPR/Cas9 cargoes have demonstrated the need to evaluate functional delivery using cargo-specific criteria, thereby encouraging the development of next-generation functional reporter systems [102]. Functional reporter systems developed for this purpose include Cre-loxP-based reporter models, split-luciferase (HiBiT/LgBiT NanoLuc) systems, miRNA-targeting reporters, and CRISPR/Cas9-based reporter approaches [102]. For protein cargoes, appropriately adapted split-luciferase or Cre-loxP-based reporter systems can provide strong evidence of functional delivery [103,104], whereas reporters based on target gene suppression can be used for miRNAs [105]. For CRISPR/Cas systems, the demonstration of genome-editing outcomes, such as indel formation, homology-directed repair (HDR), or base editing, provides the most reliable evidence of functional delivery [98,106]. These reporter systems enable the distinction between overall cellular internalization and biologically functional cargo delivery, thereby allowing a more reliable assessment of EV-mediated functional delivery.

In engineered MSC-EV platforms, loading efficiency, cellular internalization, and cargo-specific biological outcomes are being evaluated in increasing detail [23]. These studies provide important evidence of functional delivery through endpoints such as target gene suppression, protein activity, genome editing, and phenotypic changes [32,89,90]. Nevertheless, direct quantitative monitoring of the amount, structural integrity, biological half-life, and activity over time of the delivered cargo within recipient cells remains an emerging area of research. The integrated assessment of overall EV internalization, endosomal localization, cytosolic cargo access, and biological activity will contribute to a more comprehensive characterization of MSC-EV-mediated functional delivery.

## 4. Therapeutic Applications and Translation

Although the therapeutic effects of MSC-EVs on immunomodulation, tissue repair, and regeneration have been demonstrated in numerous preclinical studies, recent research has increasingly focused on engineering these vesicles into programmable biological carriers capable of delivering specific therapeutic cargoes to target cells [5,23]. Approaches such as genetic modification at the donor cell level, post-isolation cargo loading strategies, and surface engineering aim to enhance the therapeutic efficacy of MSC-EVs and direct them more selectively toward disease-specific molecular targets [23,107].

**Figure 3:**
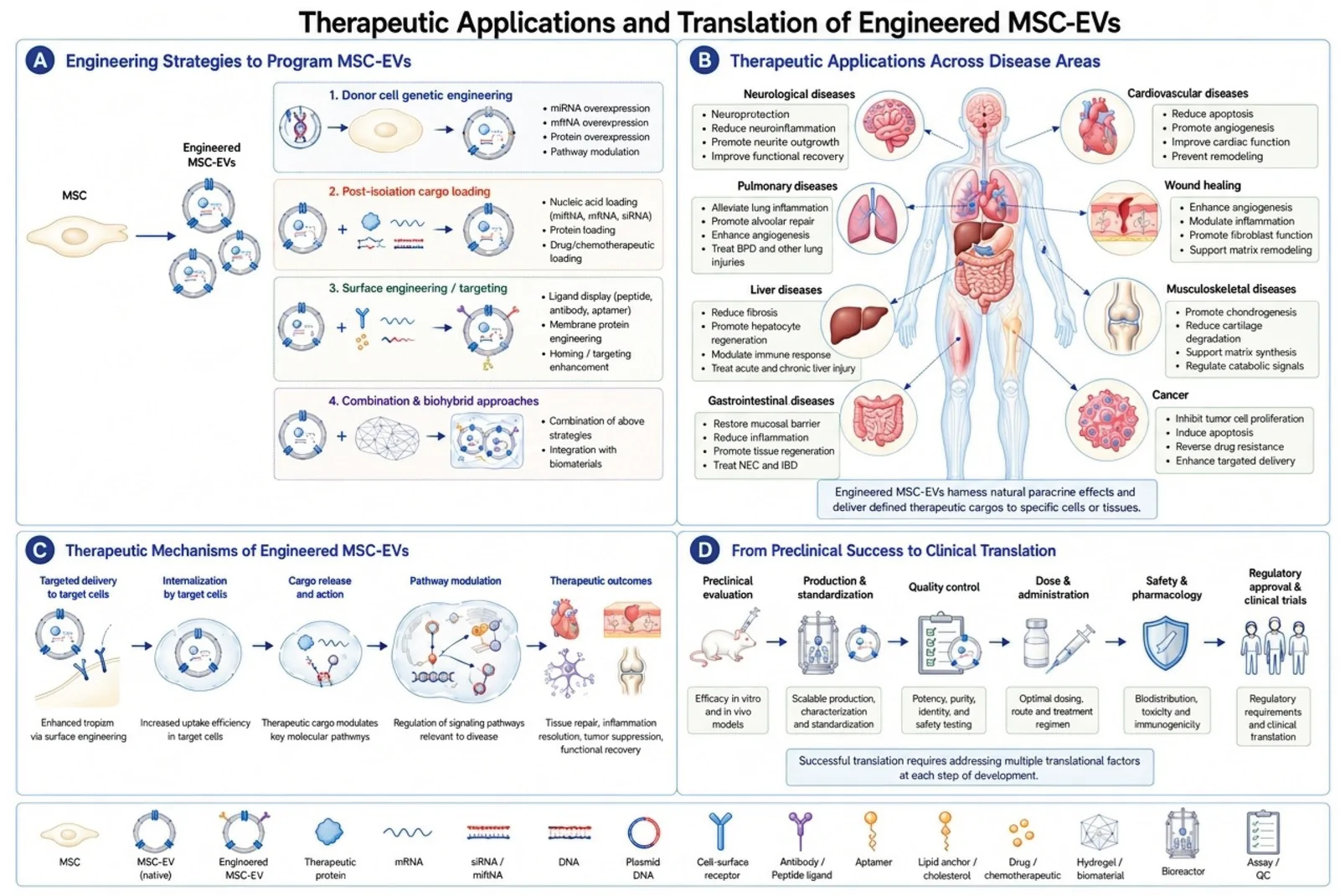
Therapeutic applications and translational framework of engineered mesenchymal stem cell-derived extracellular vesicles (MSC-EVs). (**A**) Major engineering strategies used to generate therapeutically programmed MSC-EVs, including donor-cell genetic modification to enrich selected miRNAs, mRNAs, or proteins; post-isolation loading of nucleic acids, proteins, and small-molecule drugs; surface engineering to enhance cell- or tissue-specific targeting; and combinatorial approaches integrating EV engineering with biomaterials. (**B**) Representative therapeutic applications of engineered MSC-EVs across disease areas, including cardiovascular, neurological, pulmonary, hepatic, gastrointestinal, musculoskeletal, and malignant diseases, as well as wound healing. Depending on the therapeutic cargo and engineering strategy, these platforms can modulate processes such as inflammation, apoptosis, angiogenesis, tissue regeneration, extracellular matrix remodeling, and disease-associated molecular signaling. (**C**) Conceptual sequence underlying the therapeutic action of engineered MSC-EVs, encompassing targeted delivery and enhanced interaction with recipient cells, cellular internalization, intracellular cargo release and accessibility, modulation of disease-relevant molecular pathways, and subsequent therapeutic responses. (**D**) Key stages and requirements for translation of engineered MSC-EV platforms from preclinical studies to clinical application, including preclinical efficacy assessment, scalable and standardized production, product characterization and quality control, optimization of dose and administration, evaluation of biodistribution and safety, and fulfillment of regulatory and clinical requirements. The illustration was conceptually designed by the authors and created using FigureLabs AI (https://figurelabs.ai, accessed on 1 October 2026).

To date, engineered MSC-EV platforms have shown promising results in numerous preclinical models, particularly those of cardiovascular diseases [108], wound healing [32], neurological disorders [109], cancer [12], musculoskeletal disorders [110,111], and various inflammatory diseases [112]. However, the engineering strategies, types of cargo delivered, and mechanisms of therapeutic action vary considerably across different diseases [23]. Furthermore, translating preclinical success into clinical application requires the integrated consideration of multiple translational factors, including product standardization, manufacturing processes, quality control, dosing, safety, and regulatory requirements [7,113]. In this context, this section examines the therapeutic applications of engineered MSC-EVs across different disease areas, the engineering strategies employed, and the available evidence regarding their preclinical efficacy (Figure 3, Table 4).

### 4.1. Therapeutic Applications

The primary goal of therapeutic applications of engineered MSC-EVs is to increase the amount of cargo targeting a specific disease process, tissue selectivity, or local bioavailability while preserving the regenerative and immunomodulatory properties of native vesicles [23]. To this end, various approaches have been employed, including the overexpression of therapeutic miRNAs, mRNAs, or proteins in donor MSCs [32,110,125]; the loading of nucleic acids, proteins, or drugs into isolated EVs [12,14,123]; modification of the EV surface with targeting ligands; and the integration of EVs with biomaterials [108,117,126]. Preclinical evidence indicates that engineered MSC-EV platforms can enhance therapeutic efficacy across different disease models [23]. However, therapeutic outcomes vary depending on the engineering approach used, the nature of the therapeutic cargo delivered, and the molecular signaling pathways modulated in the target disease [127].

To relate the therapeutic applications of engineered MSC-EVs to the intracellular delivery processes discussed in Section 3, representative preclinical studies are compared in Table 4 according to their engineering strategies, therapeutic cargoes, targeting or uptake evidence, intracellular molecular activity, and functional outcomes. In this context, cardiovascular disease is among the most intensively studied indications for engineered MSC-EVs [128]. The primary objectives of these studies include reducing cardiomyocyte apoptosis, promoting angiogenesis, limiting inflammation, and preventing ventricular remodeling [128]. For example, exosomes derived from miR-129-5p-modified bone marrow MSCs were reported to attenuate ventricular remodeling in mice with myocardial infarction by reducing HMGB1 expression and the inflammatory response [114]. Similarly, miR-214-3p-enriched MSC exosomes were shown to enhance cardiomyocyte survival by modulating the PTEN/p-AKT signaling pathway, promote angiogenesis in the peri-infarct region, and improve cardiac function following myocardial infarction [115]. In a more advanced engineering approach, the surface of miR-302-loaded BMSC exosomes was modified with a cardiomyocyte-specific peptide. This dual-engineering strategy enhanced cardiomyocyte uptake, reduced apoptosis and inflammation following ischemia–reperfusion injury, decreased infarct size, and improved cardiac function [108]. These findings indicate that, in cardiac applications, not only cargo enrichment but also cell-specific targeting strategies can further enhance therapeutic efficacy.

In wound healing, engineered MSC-EVs primarily target impaired angiogenesis, chronic inflammation, fibroblast senescence, and inadequate extracellular matrix remodeling [129]. eNOS-enriched exosomes derived from optogenetically engineered umbilical cord MSCs were shown to enhance angiogenesis, modulate the inflammatory microenvironment at the wound site, and accelerate tissue repair in a diabetic wound model [32]. In another example of genetic engineering at the donor cell level, engineered EVs derived from adipose-derived MSCs overexpressing HIF-1α were reported to promote fibroblast proliferation and migration, increase growth factor expression and extracellular matrix synthesis, and significantly accelerate diabetic wound healing by suppressing the inflammatory response [116]. In another approach, engineered exosomes loaded with miR-146a were incorporated into a silk fibroin patch for localized and controlled delivery. This system suppressed NF-κB-mediated inflammatory signaling by targeting IRAK1, improved macrophage polarization, and accelerated diabetic wound closure [117]. These examples demonstrate that integrating different bioengineering strategies with MSC-EV platforms can facilitate the development of more effective and controlled therapeutic approaches for wound healing.

Engineered MSC-EVs are also being increasingly investigated in models of neurological diseases [118,119]. Engineering strategies in this field include the enrichment of EVs with neuroprotective miRNAs, as well as modification of the EV surface with ligands that enhance neuronal targeting. For example, EVs derived from bone marrow MSCs genetically engineered with RVG-Lamp2b and miR-21-5p expression plasmids were reported to exhibit enhanced neuronal targeting and modulate the PTEN/Akt/mTOR signaling pathway in a cerebral ischemia model [118]. These EVs reduced neuronal injury associated with excessive autophagy by modulating the PTEN/Akt/mTOR signaling pathway and promoted neurological recovery [118]. Similarly, exosomes derived from miR-133b-modified adipose-derived MSCs reduced RhoA expression in a spinal cord injury model while enhancing the axonal regeneration-associated p-CREB/CREB and p-STAT3/STAT3 signaling pathways and increasing the levels of NF, GAP-43, GFAP, and MBP, thereby contributing to improved neurological function [119].

Musculoskeletal disorders, particularly osteoarthritis and cartilage regeneration, provide some of the clearest therapeutic examples of miRNA engineering at the donor cell level [130]. Engineered exosomes generated by overexpressing miR-140-5p in human synovial MSCs suppressed extracellular matrix loss induced by exosomal Wnt5a/Wnt5b through the noncanonical Wnt/YAP signaling pathway via RalA, thereby promoting chondrocyte proliferation and migration while also preventing cartilage degeneration in a rat model of osteoarthritis [110]. Similarly, miR-92a-3p-enriched MSC exosomes were shown to enhance chondrogenic differentiation and the expression of cartilage matrix genes by directly targeting WNT5A, thereby reducing cartilage degradation in osteoarthritis [120]. These studies suggest that enriching MSC-EVs with cartilage-specific miRNAs may represent a strategy for preserving the chondrocyte phenotype and reducing catabolic matrix degradation.

Engineered MSC-EVs have also been explored as disease-specific therapeutic platforms in the context of SARS-CoV-2 infection and COVID-19. In one strategy, MSCs were engineered to express ACE2 fused to an S-palmitoylation-dependent plasma membrane-targeting sequence, resulting in EVs enriched with ACE2 on their surface [121]. These engineered EVs exhibited enhanced binding to the SARS-CoV-2 spike receptor-binding domain, inhibited its interaction with cellular ACE2, and reduced viral infection and associated lung inflammation in preclinical models. In another approach, MSC-derived exosomes were engineered by combining miR-486-5p enrichment with SARS-CoV-2 S-RBD surface modification to enhance targeting of ACE2-expressing lung cells [122]. In a radiation-induced lung injury model, these engineered exosomes exhibited prolonged pulmonary retention and attenuated lung injury and fibrosis, while miR-486-5p-enriched MSC exosomes also reduced inflammatory cytokine levels and suppressed ferroptosis through modulation of SMAD2/Akt signaling. Together, these studies illustrate how disease- or tissue-specific molecular interactions can be incorporated into MSC-EV engineering to enhance targeting and modulate inflammatory tissue injury.

In cancer, MSC-EVs are being investigated primarily as engineered biological carriers for the delivery of defined antitumor cargoes to target cells, rather than for their natural paracrine effects [131]. In one example of chemotherapeutic delivery using MSC-EVs, exosomes derived from MSCs preincubated with paclitaxel were shown to carry the drug and inhibit tumor cell proliferation in vitro [123]. In a more advanced approach, doxorubicin was loaded into MSC exosomes by electroporation, and the exosome surface was functionalized with a MUC1 aptamer to enable selective delivery of the drug to MUC1-positive colorectal cancer cells, resulting in greater antitumor activity than free doxorubicin [18]. Furthermore, miR-20a-3p-enriched bone marrow MSC-EVs were reported to enhance the TRAIL-mediated apoptotic response by targeting c-FLIP in hepatocellular carcinoma cells and to suppress tumor growth [124]. However, because MSC-EVs may promote proliferation, angiogenesis, or immune evasion in some tumor models, the inclusion of unloaded EV, free cargo, and non-targeting RNA controls is particularly important in anticancer applications [131,132].

Across these indications the gains come from three levers: more cargo, improved targeting of recipient cells, and longer local residence. These bioengineering strategies support the development of MSC-EVs as next-generation cell-free therapeutic platforms that can be tailored to disease-specific requirements.

### 4.2. Clinical Translation

The clinical translation of engineered MSC-EVs requires not only the demonstration of therapeutic efficacy but also the development of GMP-compliant, scalable, and reproducible manufacturing processes [133]. One of the most advanced examples of clinical translation is the iExoKras^G12D^ platform, developed for metastatic pancreatic ductal adenocarcinoma harboring the KRAS^G12D^ mutation [134,135]. In this approach, exosomes produced from bone marrow-derived mesenchymal stem cells obtained from healthy donors and cultured under GMP conditions were isolated, and siRNA targeting KRAS^G12D^ was loaded into the vesicles by electroporation. In preclinical safety assessments, repeated intravenous administration did not result in evident toxicity or significant histopathological changes in either mice or rhesus macaques. Based on these manufacturing and safety data, the iEXPLORE Phase I trial (NCT03608631) evaluated intravenous administration of iExoKras^G12D^ in patients with advanced metastatic KRAS^G12D^-mutant pancreatic cancer. The treatment was reported to be well tolerated, with no dose-limiting toxicities observed and the maximum tolerated dose not reached even at the highest dose tested [134,135]. Another example of the clinical translation of engineered MSC-EVs is the Phase I/II trial NCT06896747 [136]. The study evaluated exosomes derived from mechanically preconditioned umbilical cord MSCs for the treatment of thin endometrium associated with severe intrauterine adhesions. Exosomes derived from UC-MSCs exposed to biomechanical stress during in vitro expansion (ME-UCMSC-Exo) were compared with UCMSC-derived exosomes produced under standard culture conditions and autologous platelet-rich plasma (PRP). Exosome preparations at a concentration of approximately 5 × 10^10^ particles/mL were administered to the endometrium under hysteroscopic guidance during the proliferative phase, and endometrial thickness and clinical pregnancy were evaluated. This study represents an important clinical trial in which EVs with biological properties modified through mechanical preconditioning of donor MSCs are directly compared with conventional MSC-EVs [136]. In addition to engineered MSC-EVs, native MSC-EV products are being evaluated increasingly in clinical trials. According to ClinicalTrials.gov records, native MSC-EV products have reached a more advanced stage of clinical development (Table 5). In this context, the bone marrow MSC-derived ExoFlo product has been evaluated in a completed Phase II trial and is currently being investigated in a Phase III clinical trial for ARDS [137], while umbilical cord MSC-derived EVs are being investigated in early-phase clinical trials for knee osteoarthritis [138], decompensated liver cirrhosis, and various other diseases [139]. Although these studies do not evaluate engineered products, they provide a foundation for the future clinical development of engineered MSC-EV platforms in terms of GMP-compliant manufacturing, quality control, dosing, and safety.

Dosing, safety, and biodistribution also remain areas that have not yet been fully standardized for clinical translation [7]. Across studies, EV dose is defined based on particle number, total EV protein, or the amount of loaded cargo [179]. In the first-in-human Phase I iExoKras^G12D^ trial, dosing was determined based on the amount of exosomal protein, and the starting dose was calculated from toxicity studies conducted in mice and rhesus macaques [134,135]. In this study, no dose-limiting toxicities were observed, and systemic administration was reported to have an acceptable safety profile. Biodistribution studies conducted using the same platform showed that labeled EVs reached the pancreas and liver, while analyses of clinical samples demonstrated suppression of KRAS^G12D^ and downstream ERK signaling. However, because current clinical experience is largely limited to a single siRNA-loaded MSC-EV platform, these findings cannot be directly generalized to other engineered MSC-EV products [12,134,135].

Finally, maintaining long-term stability and reliably assessing therapeutic potency are key requirements for the clinical development of engineered MSC-EV products [113]. Although trehalose-assisted lyophilization has been shown to preserve both particle characteristics and enzymatic activity in enzyme-loaded MSC-EVs, these findings require further validation for other cargo types [180]. Similarly, although CD73 activity and cell-based immunomodulatory assays have been investigated as potential approaches for assessing the potency of native MSC-EV preparations, CD73 activity alone may not reliably reflect their immunomodulatory potency [181]. For engineered products, therapeutic potency should therefore be verified using cargo-specific functional assays. Overall, current evidence indicates that the clinical development of engineered MSC-EVs is feasible; however, standardized criteria for manufacturing, quality control, dosing, stability, and functional potency have not yet been fully established across different cargo types and engineering strategies.

## 5. Conclusions and Future Perspectives

MSC-derived EVs have emerged as a growing area of research because they not only serve as natural vesicular systems for the intercellular transport of biologically active molecules but can also be engineered for therapeutic applications [182]. The studies reviewed here demonstrate that MSC-EVs can be used to deliver various therapeutic cargoes, including proteins, nucleic acids, and small molecules, and that EV engineering can influence loading capacity, target cell interactions, and biological activity [183]. However, it is clear that the therapeutic potential of EVs should not be evaluated solely on the basis of successful loading of the target molecule into the vesicle. Cellular uptake of the cargo, intracellular trafficking, escape from endosomal compartments, preservation of its biological integrity and function, and ultimately the induction of the intended cellular response are key components of a successful EV-based delivery system [61]. Within this framework, determining the therapeutic efficacy of engineered MSC-EVs requires consideration of how the applied engineering strategy affects both EV delivery to the target cell and the functional activity of the therapeutic cargo within the cell [184]. Therefore, future studies should focus on comparatively evaluating not only the cargo delivery characteristics of MSC-EVs generated using different engineering approaches but also the specific biological responses they induce in target tissues and cells.

The future therapeutic efficacy of engineered MSC-EVs is likely to depend not merely on loading different cargoes into EVs, but rather on the selective modulation of molecular targets that play critical roles in specific disease processes [110,112,114,116]. Indeed, current studies indicate that modulation of specific signaling pathways by EV-delivered cargo may provide a mechanistic basis for therapeutic efficacy. For example, miR-129-5p-enriched MSC-EVs have been shown to attenuate ventricular remodeling by reducing HMGB1 expression and the associated inflammatory response [114], while miR-146a-5p-containing EVs limited cardiomyocyte injury through MYBL1 [112], and EVs derived from HIF-1α-overexpressing MSCs promoted fibroblast proliferation and migration in diabetic wounds [116]. Together, these findings suggest that modulating specific molecular targets through EV engineering may contribute to therapeutic efficacy. Similarly, targeting Wnt5a-associated signaling pathways through miR-140-5p and miR-92a-3p in osteoarthritis demonstrates the potential of incorporating disease-specific molecular targets into MSC-EV engineering strategies [110,120].

The same principle applies to cancer therapy, although in this context the goal is to suppress molecular pathways that promote tumor cell proliferation, survival, and invasiveness [131]. For example, miR-122-modified adipose-derived MSC-EVs increased chemosensitivity [10], while doxorubicin-loaded MSC-EVs functionalized with a MUC1 aptamer were directed toward MUC1-positive cancer cells, and EVs carrying KRAS^G12D^-targeting siRNA suppressed oncogenic KRAS signaling [12,18]. Based on these findings, future studies may benefit from focusing on the identification of molecularly defined targets, selection of cargoes appropriate for these targets, and matching of target cell-specific EV surface properties with the selected cargo, rather than investigating the overall efficacy of a single cargo according to disease or tumor type. In particular, the combined design of targeting ligands with disease-specific RNA or protein cargoes would enable target cell delivery, intracellular cargo release, and functional modulation of the targeted molecular pathway to be evaluated within the same system.

The field has answered which cargoes can be loaded. The open question is which molecular target, in which cell type, is worth modulating, and that question is answered per disease, not per vesicle. This approach may help establish a direct relationship among the engineering strategy, molecular target, and biological response, thereby improving comparability across preclinical studies and facilitating the development of more rationally designed EV-based therapeutic platforms.

## Figures and Tables

**Table 1:** Source-dependent characteristics of extracellular vesicles for therapeutic cargo delivery.

Characteristic	MSC-EVs	HEK293-EVs	Macrophage-EVs	RBC-EVs	Milk-EVs
Intrinsic biological activity	Immunomodulatory and angiogenic activities; tissue-repair effects reported	Source-dependent; not primarily selected for regenerative activity	Phenotype-dependent inflammatory or immunoregulatory activity	Erythrocyte-associated biological components; regenerative activity not a defining feature	Endogenous bioactive cargo; biological effects remain context-dependent
Alignment with therapeutic objectives	Regeneration, tissue repair and inflammatory regulation	Engineered RNA and protein delivery	Immune modulation and inflammation-related applications	RNA delivery and gene-editing applications	Oral delivery and gastrointestinal applications
Endogenous molecular cargo	Proteins and regulatory RNAs associated with angiogenic and immune responses	Cellular proteins and RNAs; composition affected by producer-cell engineering	Immune-related proteins and RNAs; phenotype-dependent composition	Erythrocyte-associated proteins and RNAs	Proteins, lipids and RNAs; composition influenced by source and processing
Therapeutic cargo loading	RNA, proteins and small molecules have been loaded	RNA and protein loading demonstrated	RNA and drug loading demonstrated	ASO, mRNA and guide RNA loading demonstrated	Drug and nucleic acid loading investigated
Targeting properties	Cellular interactions and engineered targeting reported; no established universal tissue specificity	Engineered targeting ligands can be introduced	Inflammation-associated interactions investigated; phenotype- and model-dependent	Engineered targeting approaches reported	Oral uptake investigated; systemic tissue specificity remains uncertain
Scalability	Cell expansion and bioreactor production feasible; donor and passage limitations	Expandable cell lines support process development	Producer-cell differentiation and phenotype control add complexity	Blood-derived starting material; experimental large-scale EV generation demonstrated	Abundant starting material; isolation yield and purity remain challenges
Reproducibility	Affected by donor, tissue source, passage and culture conditions	Stable cell lines facilitate process standardization; EV batch consistency still requires validation	Affected by donor, phenotype and culture conditions	Dependent on blood sourcing, vesicle generation and isolation	Dependent on milk source, processing and purification

Note: This table summarizes characteristics reported for EVs from different biological sources. Cargo-loading efficiency, targeting, biological activity, and manufacturing performance depend on producer-cell characteristics, isolation methods, engineering strategies, and experimental conditions. Abbreviations: MSC-EVs, mesenchymal stromal/stem cell-derived extracellular vesicles; HEK293-EVs, human embryonic kidney 293 cell-derived extracellular vesicles; RBC-EVs, red blood cell-derived extracellular vesicles; ASO, antisense oligonucleotide.

**Table 2:** Comparative characteristics of engineering strategies for therapeutic cargo loading and targeting of MSC-derived extracellular vesicles.

Engineering Strategy	Core Approach	Commonly Applicable Cargo	Major Advantages	Major Limitations and Risks	Potential Impact on EV Integrity and Properties	Biological Problem Addressed
Endogenous (pre-isolation) loading	Genetic or biological modification of donor cells to promote cargo expression and incorporation into EVs during biogenesis	miRNAs siRNAs mRNAs lncRNAs Proteins	Enables cargo incorporation during EV biogenesis Suitable for biologically active macromolecules Avoids post-isolation physical disruption of the EV membrane	Cargo enrichment may be incomplete or heterogeneous Requires donor-cell manipulation Production may be influenced by donor-cell state and culture conditions	Generally, avoids direct post-isolation membrane disruption; however, donor-cell engineering may alter EV composition, surface characteristics, and biological activity	Enables enrichment of cargo that is difficult to introduce into isolated EVs
Endogenous active sorting	Directing cargo to EVs through engineered scaffold proteins, tetraspanin-associated systems, protein–protein interactions, or inducible sorting mechanisms	Proteins RNA Gene-editing components	Can improve the selectivity and efficiency of cargo incorporation compared with passive endogenous loading. Enables controlled cargo enrichment	Engineering systems can be complex Fusion proteins or sorting motifs may alter cargo structure or function	Avoids post-isolation physical permeabilization, but engineered EV-associated proteins may modify EV surface properties or native interactions	Promotes selective incorporation of a defined cargo during EV biogenesis
Passive incubation/diffusion	Incubation of isolated EVs with cargo to promote passive association or incorporation, particularly for membrane-permeable or hydrophobic molecules	Small hydrophobic molecules Selected drugs Other membrane-compatible cargos	Simple, relatively mild, and requires limited specialized equipment	Often associated with variable or limited loading A distinction must be made between the attachment of the cargo to the surface and true intravesicular encapsulation	Usually avoids deliberate membrane permeabilization, although cargo–membrane interactions may alter EV surface properties	Provides a simple approach for incorporating membrane-compatible cargo into isolated EVs
Electroporation	Transient electrical permeabilization of the EV membrane to facilitate cargo entry	siRNAs miRNAs mRNAs Plasmid DNA Proteins Selected small molecules	Rapid and broadly applicable Particularly useful for nucleic acid loading Readily adaptable to different cargo/EV ratios	Electroporation conditions require optimization Excessive electrical stress may alter EV properties Nucleic acid aggregation can result in overestimation of apparent loading	Membrane resealing may occur after treatment. However, inappropriate electrical parameters may affect membrane structure, size distribution, surface properties, or colloidal stability	Facilitates introduction of hydrophilic or relatively large cargo that does not efficiently cross the EV membrane
Sonication	Transient membrane deformation/permeabilization via ultrasonic energy	Proteins Nucleic acids Selected small molecules or drugs	Can provide relatively high apparent loading and is applicable to diverse cargo types	Excessive sonication may cause membrane disruption, aggregation, or changes in cargo properties Processing parameters are highly influential	Mechanical stress may alter membrane organization, morphology, size distribution, and other physicochemical properties if not carefully controlled	Facilitates loading of cargo that is poorly incorporated by passive approaches
Chemical permeabilization	Temporary increase in membrane permeability using membrane-active chemical agents	Proteins Nucleic acids Selected small molecules	Can facilitate cargo entry without specialized physical equipment Potentially useful for cargo that is poorly loaded by passive incubation	Chemical agents may affect membrane lipids or proteins Residual reagents must be removed Optimization is cargo- and EV-dependent	May directly affect membrane composition, permeability, stability, and native EV surface properties	Facilitates entry of cargo that cannot efficiently cross the intact EV membrane
Freeze–thaw cycling	Repeated freezing and thawing to induce transient membrane perturbation and facilitate cargo incorporation	Proteins Nucleic acids Selected small molecules	Simple and requires minimal specialized equipment Can facilitate incorporation of some exogenous cargos	Repeated cycles may promote EV aggregation and cargo degradation	Excessive or repeated cycles may compromise membrane integrity, colloidal stability, and cargo stability	Provides a simple physical approach for introducing exogenous cargo into isolated EVs
Lipid conjugation/membrane anchoring	Chemical conjugation of cargo to hydrophobic lipid moieties to promote association with the EV membrane	Particularly applicable to siRNAs, miRNAs, and other lipid-compatible nucleic acid cargos	Promotes efficient association with EV membranes Can improve loading of otherwise poorly membrane-associated nucleic acids	Requires chemical modification of the cargo Conjugation may alter cargo behavior, stability, biodistribution, or intracellular processing	Generally, avoids mechanical disruption of the EV membrane, but may modify membrane composition and surface characteristics	Promotes the association of hydrophilic nucleic acids with EV membranes
Surface ligand engineering	Addition or presentation of targeting ligands, including peptides, antibodies, aptamers, proteins, or other recognition molecules, on the EV surface	Generally cargo-independent and compatible with multiple cargo-loading strategies	Can enhance recognition and uptake by selected cell types or tissues and may influence in vivo biodistribution	Ligand density, orientation, and conjugation efficiency require optimization Modification may alter EV interactions and biodistribution	Surface composition, charge, protein interactions, and cellular recognition may be altered	Addresses inefficient target-cell recognition and nonspecific EV uptake
PEG- or lipid-based surface modification	Modification of the EV surface using PEGylated or lipid-anchored components to alter surface properties or introduce functional groups	Primarily used to modify EV surface characteristics, reduce nonspecific interactions, or introduce functional groups for subsequent targeting	Enables modulation of surface charge, ligand presentation, and potentially circulation-related properties	Modification density and molecular architecture require optimization Excessive modification may interfere with native EV–cell interactions	Can substantially alter EV surface physicochemical characteristics and interactions with recipient cells	Modulates surface properties and provides a platform for targeting or other functionalization
Hybrid EV–liposome systems	Integration or fusion of EVs with liposomes or other lipid-based nanocarriers to combine EV-associated biological properties with synthetic carrier characteristics	siRNA, mRNA plasmid DNA Proteins Small molecules Multimodal cargo	May combine the biological interface of EVs with the loading capacity and formulation flexibility of lipid-based systems Potentially useful for complex or multimodal delivery	Hybridization efficiency, composition, and batch consistency require careful control Product characterization becomes more complex Manufacturing and regulatory challenges may increase	Membrane composition and physicochemical properties may differ substantially from those of native EVs Preservation of EV-associated features must be demonstrated	Addresses limitations in exogenous loading capacity and formulation flexibility of native EVs
Microfluidic and advanced hybrid engineering	Use of microfluidic and related controlled-processing approaches to assemble, load, or modify EV–lipid systems	mRNA siRNA Proteins Multimodal cargo	Provides greater control over formulation and processing parameters May improve reproducibility and facilitate scalable manufacturing	Requires specialized equipment and process optimization EV recovery and membrane integrity must be validated	Effects depend strongly on the specific microfluidic process and associated physical forces Membrane integrity must be experimentally confirmed	Addresses limitations in reproducibility, loading control, and process scalability

Note: The performance of each engineering strategy depends strongly on EV source, donor-cell state, cargo properties, cargo-to-EV ratio, processing conditions, and purification procedures. Therefore, loading efficiency or EV–cargo association alone should not be considered sufficient evidence of successful intracellular delivery. Evaluation of cargo integrity, EV integrity, intracellular localization, endosomal trafficking, endosomal escape, cytosolic accessibility, and cargo-specific biological activity is required to establish functional delivery.

**Table 3:** Evidence hierarchy for demonstrating functional intracellular delivery of EV cargo.

Cargo	Level 1: Cargo Presence/Uptake	Level 2: Intracellular Accessibility	Level 3: Functional Delivery
miRNA/siRNA	EV/cargo fluorescence; intracellular RNA detection (RT-qPCR)	Endosomal colocalization; cytosolic RNA detection or reporter-based assays	Sequence-specific target suppression (RT-qPCR/Western blot); downstream molecular or phenotypic response
mRNA	Intracellular mRNA detection	Cytosolic mRNA detection; transcript integrity assessment	Translation of delivered mRNA (e.g., GFP/luciferase expression); functional response
Protein	Fluorescence-based uptake; immunological detection	Endosomal colocalization; cytosolic or target-compartment accessibility assays	Cargo-specific activity assays; target engagement; functional rescue
Enzyme	Intracellular enzyme detection (fluorescence/Western blot)	Access to the relevant intracellular compartment or substrate	Enzymatic activity assays; substrate/product conversion; functional rescue
CRISPR/Cas	Intracellular Cas9/sgRNA detection	Nuclear localization; assessment of access to genomic DNA	Target-site editing (indels, HDR, base editing); target-gene disruption
Small-molecule drug	Intracellular drug detection (fluorescence/analytical assays)	Intracellular distribution; accessibility to the intended target	Target engagement; pathway modulation; relevant phenotypic response

Note: The evidence categories represent an increasing level of mechanistic support rather than universally mandatory experimental steps. Cargo-specific functional assays provide the strongest evidence that intracellular delivery resulted in biologically active cargo. However, observed biological effects alone do not necessarily demonstrate intracellular cargo release or direct cargo-specific activity.

**Table 4:** Engineering strategies, targeting and intracellular delivery evidence, and functional outcomes of engineered MSC-EVs in preclinical therapeutic models.

Disease Model	Engineering Strategy	Therapeutic Cargo	Targeting/Uptake Evidence	Intracellular Delivery Evidence	Functional Endpoint
Myocardial infarction [114]	BMSC miR-129-5p overexpression	miR-129-5p	Specific cellular uptake evidence not confirmed	Reduced cardiac HMGB1 expression; direct cytosolic delivery not confirmed.	Reduced inflammation, apoptosis, fibrosis and ventricular remodeling
Myocardial infarction [115]	MSC miR-214-3p overexpression	miR-214-3p	Recipient cardiomyocyte and endothelial-cell responses	PTEN 3′-UTR reporter validation; PTEN reduction and AKT activation	Increased cardiomyocyte survival, angiogenesis and cardiac repair
Myocardial I/R injury [108]	DSPE-PEG-CMP surface modification and electroporation	miR-302	Increased uptake by H9c2 cells	Increased cellular miR-302 and Ki67/YAP expression	Increased proliferation; reduced myocardial injury and infarct size
Diabetic wound [32]	Optogenetic EXPLOR loading in UCMSCs	eNOS protein	Direct cellular uptake evidence not confirmed	eNOS-associated NO production and biological activity; direct cytosolic release not established	Enhanced angiogenesis, immune microenvironment modulation and wound repair
Diabetic wound [116]	HIF-1α overexpression in ADSCs	Altered endogenous EV cargo	PKH26-labeled EV internalization by fibroblasts	PI3K/AKT-associated responses and increased growth factor/ECM expression; direct HIF-1α protein transfer not established	Increased fibroblast proliferation, migration and wound closure
Diabetic wound [117]	Engineered miRNA loading, silk-binding peptide and silk fibroin patch	miR-146a	Enhanced local EV retention through silk fibroin patch	IRAK1 targeting and inflammatory signaling modulation	Reduced inflammation; enhanced angiogenesis and wound healing
Cerebral ischemia [118]	RVG-Lamp2b surface modification and miRNA enrichment	miR-21	Brain/neuronal targeting investigated	PTEN/Akt/mTOR-associated regulation of excessive autophagy	Reduced neuronal injury and improved neurological recovery
Spinal cord injury [119]	miR-133b overexpression in ADSCs	miR-133b	Specific cellular uptake evidence not confirmed	Reduced RhoA expression; increased p-CREB/CREB and p-STAT3/STAT3 ratios	Increased NF, GAP-43, GFAP and MBP expression; improved neurological recovery
Osteoarthritis [110]	miR-140-5p overexpression in synovial MSCs	miR-140-5p	Chondrocyte responses assessed	RalA-dependent modulation of Wnt5a/Wnt5b–YAP signaling	Increased chondrocyte proliferation/migration without ECM loss; reduced OA progression
Osteoarthritis [120]	MSC miR-92a-3p overexpression	miR-92a-3p	MSC and chondrocyte responses assessed	WNT5A targeting validated by luciferase reporter assay	Enhanced chondrogenesis and cartilage matrix synthesis; reduced degradation
SARS-CoV-2 infection [121]	Palmitoylation-dependent ACE2 surface engineering	Surface-displayed ACE2	S-RBD binding and inhibition of viral attachment	Extracellular neutralization; intracellular delivery not applicable	Reduced viral infection and lung inflammation
Radiation-induced lung injury [122]	UCMSC miR-486-5p enrichment and S-RBD surface modification	miR-486-5p	Increased pulmonary retention in ACE2-humanized mice	SMAD2 3′-UTR reporter validation; SMAD2 inhibition and AKT phosphorylation	Reduced ferroptosis, lung injury and pulmonary fibrosis
Pancreatic cancer, in vitro [123]	Paclitaxel priming of MSCs; isolation of drug-containing microvesicles	Paclitaxel	Specific recipient-cell uptake mechanism not established	PTX-associated antiproliferative activity; intracellular drug release not directly demonstrated	Reduced CFPAC-1 cell proliferation
Colorectal cancer [18]	Electroporation and MUC1 aptamer conjugation	Doxorubicin	Preferential delivery to MUC1-positive cells and increased tumor accumulation	Drug-associated cytotoxic activity; endosomal escape not established	Reduced tumor growth compared with free doxorubicin
Hepatocellular carcinoma [124]	BMSC miR-20a-3p overexpression	miR-20a-3p	Recipient HCC-cell responses evaluated	c-FLIP targeting and rescue experiments; increased TRAIL-related signaling	Increased apoptosis and reduced tumor growth

Note: Targeting and uptake evidence includes tissue accumulation, local retention, and cellular internalization, which represent distinct stages of delivery. Intracellular delivery evidence refers to reported molecular changes consistent with cargo activity, including target gene regulation, signaling pathway modulation, and enzymatic responses. Abbreviations: ADSC, adipose-derived stem cell; BMSC, bone marrow mesenchymal stem cell; CMP, cardiomyocyte-specific peptide; ECM, extracellular matrix; eNOS, endothelial nitric oxide synthase; EV, extracellular vesicle; I/R, ischemia–reperfusion; MSC, mesenchymal stem cell; OA, osteoarthritis; PTX, paclitaxel; UCMSC, umbilical cord mesenchymal stem cell.

**Table 5:** Registered clinical studies of MSC-derived extracellular vesicle products identified in ClinicalTrials.gov.

	Registry ID	Registration Date	Disease	MSC Source	Engineered/Unmodified	Phase	Route	Status	Location	Reference
**1**	NCT03608631	16 July 2018	Metastatic pancreatic ductal adenocarcinoma with KRAS^G12D^ mutation	Bone marrow MSC	Engineered	Phase I/II	Intravenous	Recruiting	USA	Kalluri et al., Nature Communications, 2025 [134,135]
**2**	NCT06896747	18 March 2025	Thin endometrium/intrauterine adhesions	Umbilical cord MSC	Engineered; unmodified comparator included	Phase I/II	Hysteroscopy-guided intra-endometrial injection	Recruiting	China	ClinicalTrials.gov: NCT06896747 [136]
**3**	NCT04356300	19 April 2020	Multiple organ dysfunction after acute type A aortic dissection repair	Umbilical cord MSC	Unmodified	Not applicable	Intravenous	Unknown; last reported as not yet recruiting	China	ClinicalTrials.gov: NCT04356300 [140]
**4**	NCT05871463	7 April 2023	Decompensated liver cirrhosis	Umbilical cord MSC	Unmodified	Phase II	Intravenous	Unknown; last reported as recruiting	Iran	ClinicalTrials.gov: NCT05871463 [141]
**5**	NCT05808400	7 April 2023	Chronic cough after COVID-19	Umbilical cord MSC	Unmodified	Early Phase I	Nebulized inhalation	Unknown; last reported as recruiting	China	ClinicalTrials.gov: NCT05808400 [142]
**6**	NCT05216562	28 January 2022	Moderate COVID-19 with hyperinflammation	Not specified	Unmodified	Phase II/III	Intravenous	Unknown; last reported as recruiting	Indonesia	ClinicalTrials.gov: NCT05216562 [143]
**7**	NCT03437759	23 January 2018	Macular hole	Umbilical cord MSC	Unmodified	Early Phase I	Intravitreal administration following pars plana vitrectomy	Unknown	China	ClinicalTrials.gov: NCT03437759 [144]
**8**	NCT05523011	25 August 2022	Healthy volunteers; topical safety and tolerability	Not specified	Unmodified	Phase I	Topical ointment	Completed	Singapore	Chandran et al., Cytotherapy, 2025 [145,146]
**9**	NCT05499156	30 May 2022	Resistant perianal fistula in Crohn’s disease	Placental MSC	Unmodified	Phase I/II	Local/perifistular injection	Unknown; last reported as recruiting	Iran	ClinicalTrials.gov: NCT05499156 [147]
**10**	NCT05738629	13 February 2023	Dry eye disease after refractive surgery/blepharospasm	PSC-derived MSC	Unmodified	Phase I/II	Eye drops	Unknown	China	ClinicalTrials.gov: NCT05738629 [148]
**11**	NCT05669144	10 December 2022	Myocardial ischemia/infarction in CABG candidates	Not specified	Unmodified; combination intervention	Phase I/II	Local cardiac co-transplantation with autologous mitochondria	Unknown; last reported as recruiting	Iran	ClinicalTrials.gov: NCT05669144 [149]
**12**	NCT02138331	12 May 2014	Type 1 diabetes mellitus	Umbilical cord blood MSC	Unmodified	Phase II/III	Intravenous	Unknown; last reported as enrolling by invitation	Egypt	ClinicalTrials.gov: NCT02138331 [150]
**13**	NCT05060107	14 September 2021	Knee osteoarthritis (ExoOA-1)	Allogeneic MSC	Unmodified	Phase I	Intra-articular	Unknown; last reported as not yet recruiting	Chile	ClinicalTrials.gov: NCT05060107 [151]
**14**	NCT06431152	17 May 2024	Knee osteoarthritis	Umbilical cord MSC	Unmodified	Early Phase I	Intra-articular	Unknown; last reported as recruiting	Chile	ClinicalTrials.gov: NCT06431152 [152]
**15**	NCT03384433	29 November 2017	Acute ischemic stroke	Allogeneic MSC; tissue source not specified	Unmodified	Phase I/II	Stereotactic/intraparenchymal	Unknown; last reported as recruiting	Iran	ClinicalTrials.gov: NCT03384433 [153]
**16**	NCT06598202	10 September 2024	Amyotrophic lateral sclerosis	Human umbilical cord blood MSC	Unmodified	Phase I/II	Intranasal drops	Recruiting	China	ClinicalTrials.gov: NCT06598202 [154]
**17**	NCT04173650	18 November 2019	Dystrophic epidermolysis bullosa	Bone marrow MSC	Unmodified	Phase I/IIa	Topical wound application	Recruiting	United States	ClinicalTrials.gov: NCT04173650 [155]
**18**	NCT05402748	25 May 2022	Complex non-Crohn’s perianal fistula	Placental MSC	Unmodified	Phase I/II	Injection into the fistula tract	Unknown; last reported as recruiting	Iran	ClinicalTrials.gov: NCT05402748; IRCT20211212053361N1 [156]
**19**	NCT04491240	27 July 2020	COVID-19-associated pneumonia	MSC source not explicitly specified	Unmodified/MSC origin incompletely reported	Phase I/II	Inhalation	Completed	Russian Federation	ClinicalTrials.gov: NCT04491240 [157]
**20**	NCT04388982	28 April 2020	Mild-to-moderate Alzheimer’s disease	Adipose MSC	Unmodified	Phase I/II	Intranasal	Unknown; primary results published	China	Xie et al., General Psychiatry, 2023 [158]
**21**	NCT06812637	2 February 2025	Diabetic foot ulcer	Wharton’s jelly MSC	Unmodified	Phase I	Topical exosome gel	Completed	Egypt	Kishta et al., Stem Cell Research & Therapy, 2025 [159,160]
**22**	NCT06919380	13 February 2025	Anti-MDA5-positive dermatomyositis-associated rapidly progressive interstitial lung disease	Not specified	Unmodified	Phase I	Nebulization	Recruiting	China	ClinicalTrials.gov: NCT06919380 [161]
**23**	NCT04493242	29 July 2020	COVID-19-associated moderate-to-severe ARDS	Bone marrow MSC	Unmodified	Phase II	Intravenous	Completed	United States	Lightner et al., CHEST, 2023 [162,163]
**24**	NCT05127122	9 November 2021	Acute respiratory distress syndrome	Bone marrow MSC	Unmodified	Phase I/II	Intravenous	Withdrawn before enrollment	United States	ClinicalTrials.gov: NCT05127122 [164]
**25**	NCT05354141	27 April 2022	Moderate-to-severe acute respiratory distress syndrome	Bone marrow MSC	Unmodified	Phase III	Intravenous	Recruiting	United States	ClinicalTrials.gov: NCT05354141 [165]
**26**	NCT04657458	7 December 2020	COVID-19-associated moderate-to-severe ARDS	Bone marrow MSC	Unmodified	Expanded access	Intravenous	No longer available	United States	Sengupta et al., Stem Cell Reviews and Reports, 2026 [166,167]
**27**	NCT05176366	17 November 2021	Medically refractory ulcerative colitis	Bone marrow MSC	Unmodified	Phase I	Intravenous	Terminated	United States	ClinicalTrials.gov: NCT05176366 [168]
**28**	NCT07144241	20 August 2025	Type 2 diabetes mellitus	Not specified; Exo-HWS product	Unmodified; combination arm includes HUVEC-derived exosomes	Phase II	Intravenous bolus injection, monthly for 3 months	Not yet recruiting	Indonesia	ClinicalTrials.gov: NCT07144241 [169]
**29**	NCT07146087	20 August 2025	Progressive multiple sclerosis	Allogeneic bone marrow MSC	Unmodified	Early Phase I	Intravenous infusion every 12 weeks; 4 infusions	Suspended because of slow enrollment	Poland	ClinicalTrials.gov: NCT07146087 [170]
**30**	NCT07243561	13 November 2025	Childhood autism spectrum disorder	Human umbilical cord MSC	Unmodified	Phase I/II	Intranasal spray	Recruiting	China	ClinicalTrials.gov: NCT07243561 [171]
**31**	NCT07312656	5 December 2025	Radiation-induced oral mucositis in patients receiving radiotherapy for head and neck cancer	Not specified	Unmodified	Phase II	Oral mucosal spray	Not yet recruiting	China	ClinicalTrials.gov: NCT07312656 [172]
**32**	NCT07373054	30 December 2025	Androgenetic alopecia	Human umbilical cord MSC	Unmodified; lyophilized formulations compared	Not applicable	Automated microneedling-assisted scalp delivery	Recruiting	China	ClinicalTrials.gov: NCT07373054 [173]
**33**	NCT07485751	16 March 2026	Negative symptoms of treatment-resistant schizophrenia	Human dental follicle MSC	Unmodified	Early Phase I	Intranasal spray	Not yet recruiting	Location not reported in registry	ClinicalTrials.gov: NCT07485751 [174]
**34**	NCT07508033	27 March 2026	Alveolar ridge preservation after tooth extraction	MSC source not specified	Unmodified	Phase I/II	Local application into the extraction socket	Recruiting	Location not reported in registry	ClinicalTrials.gov: NCT07508033 [175]
**35**	NCT07510139	22 March 2026	Premature ovarian insufficiency	Umbilical cord MSC	Unmodified	Phase I/II	Intra-ovarian injection	Active, not recruiting	China	ClinicalTrials.gov: NCT07510139 [176]
**36**	NCT07519031	1 April 2026	Non-segmental vitiligo	Human umbilical cord MSC	Unmodified	Phase I/II	Intravenous infusion for progressive disease; local intralesional injection for stable disease	Not yet recruiting	China	ClinicalTrials.gov: NCT07519031 [177]
**37**	NCT07693036	8 June 2026	Type 2 diabetes mellitus	Human umbilical cord MSC	Unmodified	Phase I/II	Intravenous infusion once weekly for 8 weeks	Recruiting	China	ClinicalTrials.gov: NCT07693036 [178]

ClinicalTrials.gov was last searched on 6 August 2026. The official ClinicalTrials.gov record is cited for all studies; where available, the peer-reviewed primary results publication is also cited.

## Data Availability

No new data were created or analyzed in this study. Data sharing is not applicable.

## Abbreviations

ARDS	Acute respiratory distress syndrome
CAP	Chondrocyte-affinity peptide
Cas9	CRISPR-associated protein 9
CPP	Cell-penetrating peptides
CRISPR	Clustered regularly interspaced short palindromic repeats
CRY2	Cryptochrome 2
CSF	Cerebrospinal fluid
DNA	Deoxyribonucleic acid
DSPE	1,2-Distearoyl-sn-glycero-3-phosphoethanolamine
EMT	Epithelial–mesenchymal transition
eNOS	Endothelial nitric oxide synthase
ERK	Extracellular signal-regulated kinase
ESCRT	Endosomal sorting complex required for transport
EVs	Extracellular vesicles
GFP	Green fluorescent protein
GMP	Good manufacturing practice
HCC	Hepatocellular carcinoma
HCP5	HLA Complex P5
UCMSC	Umbilical cord-derived mesenchymal stem cells
lncRNA	Long non-coding RNA
MSC	Mesenchymal stem cells
MSC-EV	Mesenchymal stem cell-derived extracellular vesicles
MSC-sEVs	Mesenchymal stem cell-derived small extracellular vesicles
miRNA	MicroRNA
NAFLD	Non-alcoholic fatty liver disease
NF-κB	Nuclear factor kappa B
PEG	Polyethylene glycol
Rab5	Ras-related protein Rab5
Rab7	Ras-related protein Rab7
RNA	Ribonucleic acid
RISC	RNA-induced silencing complex
sEVs	Small extracellular vesicles
siRNA	Small interfering RNA
VEGF-A	Vascular endothelial growth factor A
